# Baicalein and Baicalin Inhibit SARS-CoV-2 RNA-Dependent-RNA Polymerase

**DOI:** 10.3390/microorganisms9050893

**Published:** 2021-04-22

**Authors:** Keivan Zandi, Katie Musall, Adrian Oo, Dongdong Cao, Bo Liang, Pouya Hassandarvish, Shuiyun Lan, Ryan L. Slack, Karen A. Kirby, Leda Bassit, Franck Amblard, Baek Kim, Sazaly AbuBakar, Stefan G. Sarafianos, Raymond F. Schinazi

**Affiliations:** 1 Center for AIDS Research, Laboratory of Biochemical Pharmacology, Department of Pediatrics, Emory University School of Medicine and Children’s Healthcare of Atlanta, Atlanta, GA 30322, USA; musall.katie@gmail.com (K.M.); adrian.oo@emory.edu (A.O.); shuiyun.lan@emory.edu (S.L.); ryan.slack@emory.edu (R.L.S.); karen.kirby@emory.edu (K.A.K.); lbassit@emory.edu (L.B.); famblar@emory.edu (F.A.); baek.kim@emory.edu (B.K.); stefanos.sarafianos@emory.edu (S.G.S.); rschina@emory.edu (R.F.S.); 2 Department of Biochemistry, Emory University School of Medicine, Atlanta, GA 30322, USA; dongdong.cao@emory.edu (D.C.); bo.liang@emory.edu (B.L.); 3 Tropical Infectious Diseases Research and Education Center, Department of Medical Microbiology, Faculty of Medicine, University of Malaya, Kuala Lumpur 50603, Malaysia; pouya3132@gmail.com (P.H.); sazaly@um.edu.my (S.A.); 4 Center for Drug Discovery, Children’s Healthcare of Atlanta, Atlanta, GA 30322, USA

**Keywords:** SARS-CoV-2, antiviral agents, baicalein, baicalin, coronavirus, COVID-19, RdRp, nsp12, flavonoid

## Abstract

Coronavirus Disease 2019 (COVID-19) is a deadly emerging infectious disease caused by Severe Acute Respiratory Syndrome Coronavirus 2 (SARS-CoV-2). Because SARS-CoV-2 is easily transmitted through the air and has a relatively long incubation time, COVID-19 has rapidly developed into a global pandemic. As there are no antiviral agents for the prevention and treatment of this severe pathogen except for remdesivir, development of antiviral therapies to treat infected individuals remains highly urgent. Here, we showed that baicalein and baicalin exhibited significant antiviral activity against SARS-CoV-2, the causative agent of COVID-19 through in vitro studies. Our data through cell-based and biochemical studies showed that both compounds act as SARS-CoV-2 RNA-dependent RNA polymerase (RdRp) inhibitors directly and inhibit the activity of the SARS-CoV-2 RdRp, but baicalein was more potent. We also showed specific binding of baicalein to the SARS-CoV-2 RdRp, making it a potential candidate for further studies towards therapeutic development for COVID-19 as a selective non-nucleoside polymerase inhibitor.

## 1. Introduction

**Figure 1:**
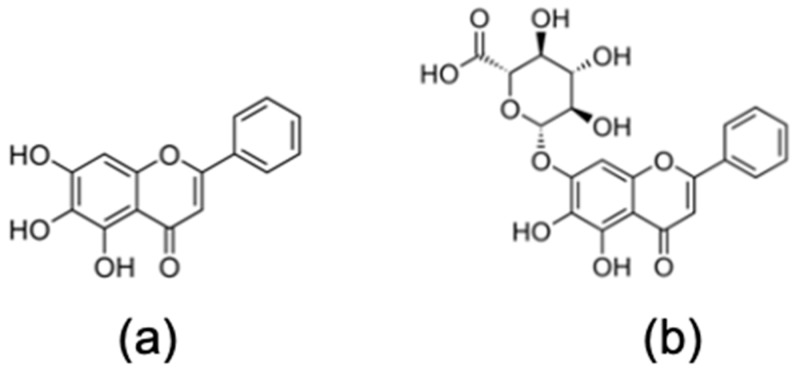
Chemical structures of (**a**) baicalein and (**b**) baicalin.

Emergence of novel viruses is a phenomenon that is common among coronaviruses (CoVs) because trafficking between species can result in novel infectious disease outbreaks. Most recently this led to the emergence of Severe Acute Respiratory Syndrome Coronavirus 2 (SARS-CoV-2), an enveloped, positive-sense single-stranded RNA virus and the causative agent of Coronavirus Disease 2019 (COVID-19), a deadly emerging infectious disease [1]. This novel single-stranded enveloped RNA virus belongs to the *Betacoronavirus* genus and is the seventh known human coronavirus [2]. Because SARS-CoV-2 is easily transmitted through the air and has a relatively long incubation time, COVID-19 has rapidly developed into a global pandemic. COVID-19 is imposing a tremendous public health threat. Thus, development of antiviral therapies to treat infected individuals remains highly urgent. The FDA recently authorized the emergency use of the drug remdesivir, which in its active triphosphate form is an RNA-dependent RNA polymerase inhibitor, for patients hospitalized with severe COVID-19 [3]. However, this drug has to be given intravenously, which restricts its use to hospital settings; it is also associated with severe side effects and appears not effective when used in early stages of the disease (https://www.who.int/emergencies/diseases/novel-coronavirus-2019/global-research-on-novel-coronavirus-2019-ncov/solidarity-clinical-trial-for-covid-19-treatments (accessed on 20 December 2020)). In the search for a quick response to the COVID-19 pandemic, the “repurposing” of approved and safe drugs is one of the fastest and most desirable approaches. In this study, we show that baicalein and baicalin (Figure 1) are potential antiviral therapeutic candidates that inhibit the in vitro replication of SARS-CoV-2 in cell culture systems by inhibiting the RNA-dependent RNA polymerase (RdRp) of SARS-CoV-2, which could give advantage to these two compounds as non-nucleoside analog SARS-CoV-2 RdRp inhibitors.

Interestingly, we have already shown in different studies that these compounds can inhibit other emerging RNA viruses such as dengue virus (DENV), Zika virus (ZIKV) and Chikungunya virus (CHIKV) [4,5,6,7,8]. Baicalein and baicalin are natural bioactive phenolic flavonoid compounds and are primarily available from the root of *Scutellaria baicalensis* Georgi (Huang Qin), an East Asian skullcap plant that contains abundant flavonoids, which are widely used in traditional Chinese medicine as a remedy for the clinical treatment of hyperlipidemia, hypertension, atherosclerosis, dysentery, common cold and inflammatory diseases [9,10]. Baicalin is metabolized to baicalein in the intestine. Interestingly, baicalein is currently evaluated in a Phase 2 clinical trial for the treatment of influenza virus infections [11].

## 2. Materials and Methods

### 2.1. Flavonoids

Baicalein and baicalin were purchased from Sigma Chemical Company (Sigma, St. Louis, MO, USA). A stock solution with concentration of 40 mM was prepared in dimethyl sulfoxide (DMSO) (Sigma-Aldrich, St. Louis, MO, USA) followed by aliquoting and storage at −20 °C until time of use. At the time of the experiment, the stock solution was diluted with MEM (Gibco, NY, USA), containing 2% fetal bovine serum (FBS) and was sterilized with a 0.2 μm syringe filter (Millipore, Burlington, MA, USA).

### 2.2. Cells and Virus

The Vero CCL-81 cell line (African Green Monkey Kidney) from ATCC (Manassas, Virginia, VA, USA) was used in this study. Vero cells were cultured in MEM containing 10% heat-inactivated fetal bovine serum (FBS) and were incubated at 37 °C in the presence 5% CO_2_. At the time of virus inoculation and antiviral assays, the concentration of FBS was reduced to 2%. SARS-CoV-2 (NR-52281: USA-WA/2020) was provided by BEI Resources (Manassas, Virginia, VA, USA) and propagated in Vero cells followed by titration using the median tissue culture infectious dose (TCID_50_) method [12]. The viral stocks were stored in aliquots at −80 °C until further use.

### 2.3. Cytotoxicity Assay

Baicalein and baicalin were evaluated for toxicity using an MTS (3-(4,5-dimethylthiazol-2-yl)-5-(3-carboxymethoxyphenyl)-2-(4-sulfophenyl)-2H-tetrazolium) reagent in Vero cells [13]. The MTS assay was performed on day 4 post-treatment in Vero cells using a CellTiter 96^®^ Non-Radioactive Cell Proliferation kit (Promega, Madison, WI, USA). Remdesivir (synthesized in our laboratory; 98% pure by LC-MS-MS) was used as a positive control with reported activity against SARS-CoV-2 [14]. Briefly, the half maximal cytotoxic concentration (CC_50_) value was determined for each compound in Vero cells.

### 2.4. Virus Yield Assay

We previously established a qRT-PCR assay specifically to quantify the yield of SARS-CoV-2 in our antiviral cell-based assay experiments [15]. Briefly, a one-step qRT-PCR was carried out containing extracted viral RNA, probe/primer mix (Forward Primer: 5′-GAC CCC AAA ATC AGC GAA AT-3′; Reverse Primer: 5′-TCT GGT TAC TGC CAG TTG AAT CTG-3′; Probe: 5′-FAM-ACC CCG CAT TAC GTT TGG TGG ACC-BHQ1-3′). Quantitative PCR measurement was performed using StepOnePlus real time PCR system (Roche, Mannheim, Germany) and the yield of progeny virus production was assessed from supernatants at interval time points using a specific q-RT PCR for SARS-CoV-2. It was observed that 48 h post-infection, a significant increase in virus yield was achieved with no observed cell death and cytopathic effect (CPE) on infected cells.

### 2.5. Antiviral Activity Assay

In order to determine the antiviral activity of the compounds, both compounds were studied in a concentration-dependent manner to identify their potencies as anti-SARS-CoV-2 agents as previously described [15]. Briefly, a monolayer of Vero cells in a 96-well cell culture microplate was treated with different nontoxic concentrations of each compound for 1 h followed by inoculation of SARS-CoV-2 at 0.1 MOI [16]. Following 1 h of adsorption incubation at 37 °C, the virus inoculum was removed and the compound- or vehicle-containing medium was added to the respected wells. The yield of progeny viruses was measured 2 days post-treatment, and infection in the supernatant of treated, infected cells by specific quantitative RT-PCR was as described above.

### 2.6. Statistical Analysis

The median effective concentration (EC_50_) and the concentration with 90% of inhibitory effect (EC_90_) were calculated using GraphPad PRISM for Windows, version 5 (GraphPad Software Inc., San Diego, CA, USA, 2005) as the mean ± standard deviation (SD) of the mean of triplicate assays from three independent experiments. The selectivity index (SI) for each effective compound was calculated as the ratio of CC_50_/EC_50_.

### 2.7. Time-of-Drug-Addition Assay

Confluent Vero cells in 96-well tissue culture plates were prepared and infected with SARS-CoV-2 at an MOI 0.1 followed by addition of the compound at the EC_90_ at different time points. Samples were analyzed by virus yield assay as described above.

### 2.8. SARS-CoV-2 Pseudovirus Entry Assay

SARS-CoV-2 pseudovirus was produced by co-transfection of 293T cells with pNL4-3 Luc R-E- and plasmids encoding either SARS-CoV-2 S K1266Stop or VSV-G (Vesicular stomatitis virus G protein) using jetPRIME (Polyplus-transfection # 114-15). Supernatants were harvested at 48 h post-transfection and filtered through a 0.45 μm filter to remove cell debris. To test the inhibition of pseudovirus entry, Huh7.5 cells in 96-well plates were pre-incubated with each compound in separate experiments for one hour followed by infection with SARS-CoV-2 pseudovirus. At 48 h post-infection, luciferase activity was measured with the Britelite plus reporter gene assay system (PerkinElmer #6066761) [17].

### 2.9. SARS-CoV-2 RdRp Assay

SARS-CoV-2 RdRp complex was prepared as described previously [15]. SARS-CoV-2 RdRp activity was measured using a reaction mixture consisting of TRIS-HCl (25 mM, pH8), NTPs (50 mM ATP, CTP and TTP; 25 mM GTP), [α-^32^P]-GTP (0.1 μM), RNA primer, RNA template, SARS-CoV-2 RdRp complex (0.1 μM) and various concentrations of each compound of interest. After incubating the reaction mixtures at 30 °C for 10 min, MnCl_2_ (5 mM) was added into each reaction. The RNA polymerase reactions were stopped after 30 min of incubation at 30 °C, by adding formamide containing 40 mM EDTA and heated at 95 °C for 10 min. Then the reaction mixture was resolved on 20% polyacrylamide-urea denaturing gels (SequaGel, National Diagnostics, Atlanta, GA, USA), and the resulting primer extended products were visualized using the Amersham Typhoon IP (Cytiva, Marlborough, MA, USA) and analyzed using the ImageQuant TL 8.2 (Cytiva) [18].

### 2.10. In Silico Study

An in silico study was performed with each compound of interest and SARS-CoV-2 RdRp. Briefly, single particle cryo-electron microscopy (cryo-EM) structures were retrieved from the Protein Data Bank (PDB), named SARS-CoV-2 RdRp/RNA complex (PDB ID: 6XQB). Both baicalin and baicalein structures and information were downloaded from PubChem website. AutoDock Vina 1.5.6 and Discovery Studio were used for ligands and receptor preparation and molecular binding study. The data from AutDock Vina software were analyzed using PyMOL and Discovery Studio 2.5.

### 2.11. Thermal Shift Assay

To assess the binding of baicalein to different SARS-CoV-2 nonstructural proteins, a thermal shift assay (TSA) was conducted as previously described by our group [19,20]. Briefly, SARS-CoV-2 nsp5, nsp12, and nsp14 proteins were expressed and purified as previously described [21,22]. Each TSA reaction contained 5 µM protein in 10 mM HEPES (pH 8.0), 6 mM MgCl_2_, 0.5 mM TCEP, 2× Sypro Orange Protein Gel Stain (Life Technologies, Carlsbad, CA, USA), and either 0.25% DMSO as control or 50 µM baicalein (0.25% DMSO final). DMSO and baicalein were incubated with protein for 20 min on ice before beginning the reaction. The plate was heated from 25 to 95 °C with a heating rate of 0.2 °C every 10 s in the QuantStudio 3 Real-Time PCR system (Thermo Fisher Scientific, Waltham, MA, USA). The intensity was measured with an Ex range of 475–500 nm and an Em range of 520–590 nm. Melting temperature (*T_m_*) of each protein, which is a measure of protein stability, was calculated using the Protein Thermal Shift^TM^ Software v1.3 (Thermo Fisher Scientific, Waltham, MA, USA). The difference in the melting temperature (Δ*T*_m_) of protein in DMSO (*T*_0_) versus in the presence of compound (*T*_m_) was calculated using the equation Δ*T*_m_ (°C) = *T*_m_ − *T*_0_. Values represent data from at least two independent experiments.

## 3. Results and Discussion

In this study, we evaluated the anti-SARS-CoV-2 activity of two natural products, baicalein and baicalin, as novel antiviral therapeutic candidates for COVID-19 through cell-based, biochemical, and biophysical studies. Of note, there is a recent publication about the effect of baicalein against SARS-CoV-2, but without any mechanistic or comprehensive in vitro investigations [23]. We have used our previously established and optimized in vitro SARS-CoV-2 cell-based virus yield assay method for SARS-CoV-2 antiviral activity using our biosafety level 3 (BSL-3) facility [15] in order to evaluate the antiviral activity of each compound against SARS-CoV-2 following measurement of cytotoxicity of the compounds against Vero cells using an MTS assay. We also used remdesivir side by side with our compounds in all cell-based assays as an established and proven control for inhibition of SARS-CoV-2 antiviral activity. As shown in Table 1, none of our tested compounds, including remdesivir, showed significant cytotoxicity in Vero cells. However, to avoid any probable toxicity from the compounds which might affect the results of cell-based antiviral studies, we used 20 µM of each compound as the highest concentration in cell-based antiviral assays.

**Figure 2:**
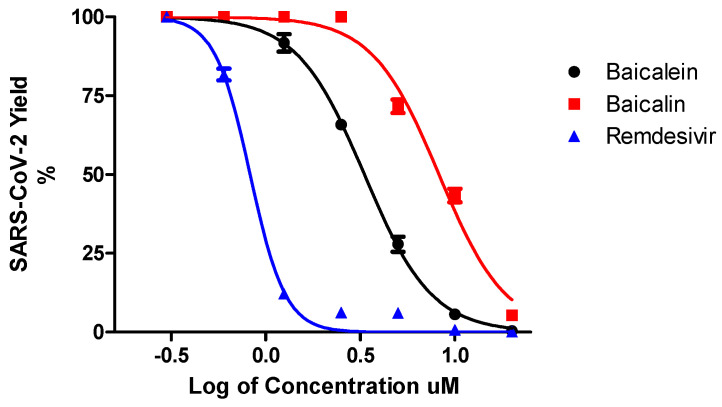
Dose-dependent antiviral study. Antiviral activity of baicalein and baicalin and remdesivir against SARS-CoV-2 in Vero cells was measured in a dose-dependent manner. SARS-CoV-2 Yield (%) was calculated based on the viral RNA copy number for each sample compared to untreated-infected control.

In the initial antiviral evaluation assay, baicalein and baicalin at 20 µM showed 99.8% and 98% inhibition of SARS-CoV-2, respectively. Therefore, both compounds were studied further to identify their potencies in a concentration-dependent manner by a virus yield reduction assay, 2 days post-treatment and infection in the supernatant of treated-infected cells. Baicalein and baicalin exhibited dose-dependent inhibitory effects against SARS-CoV-2 replication in Vero cells (Figure 2). However, baicalein exhibited more potent activity against SARS-CoV-2 compared to baicalin with an EC_50_ = 4.5 µM and EC_90_ = 7.6 µM. To confirm the antiviral activity of both compounds, we also tested them against Calu3 human lung cells, and we also demonstrated the anti-SARS-CoV-2 activity for both compounds in these cells (Table 1).

**Figure 3:**
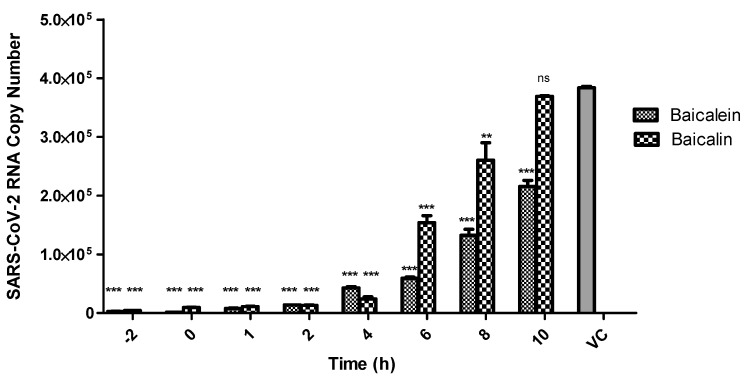
Time-of-addition assay. The effects of baicalein and baicalin on SARS-CoV-2 in vitro replication were assessed at different time points of compound addition. A single concentration of each compound based on EC_90_ value of the compound was added to the respective wells from 2 h prior infection up to 10 h post infection. Data for each time point were analyzed independently to the virus control via two-way ANOVA analysis using GraphPad Prism 7 for Windows. The results are presented as means ± standard error whereby *p* < 0.01 was represented as **, *p* < 0.001 as *** and nonsignificant readings were labelled as ns.

Next, in a time-of-addition assay, we found that baicalein inhibits the intracellular replication of SARS-CoV-2 significantly, even when added up to 8 h post-infection. However, baicalin also showed significant inhibitory effect against SARS-CoV-2 replication in Vero cells when added up to 6 h post-infection (Figure 3).

In the time-of-addition assay, both compounds showed significant effects on early stages of SARS-CoV-2 replication through a 6–8 h post-infection. Therefore, in the next step towards revealing the mechanism of action of the tested compounds we used a SARS-CoV-2 pseudovirus system to evaluate the effect of the compounds on SARS-CoV-2 cell entry. Our results from the SARS-CoV-2 pseudovirus entry assay showed that there was no inhibitory activity of baicalein or baicalin in this step of virus replication (Table 2). As the data from our time-of-addition study confirmed the anti-SARS-CoV-2 activity of our compounds of interest up to 6–8 h post infection, it could be hypothesized that the observed antiviral effects could be due to inhibiting vital viral enzymes such as the RdRp that are essential for SARS-CoV-2 replication. Therefore, we tested the effects of both compounds against the polymerization activity of SARS-CoV-2 RdRp using a biochemical assay. The SARS-CoV-2 RNA polymerase complex (nsp12/nsp7/nsp8), which was expressed and purified from insect cells, extends a 4-mer RNA primer (5′-ACGC) annealed to a 14-mer RNA template (5′-UUAUUUGUUCGCGU). In this assay, the first nucleotide to be incorporated by the polymerase is [α-^32^P]-GTP (opposite “C” in template), which allows visualization of the extended RNA products with rNTPs after resolving on a urea-denaturing gel. The amounts of the fully extended RNA products have been used for measuring the biochemical inhibitory efficacy of the proposed compounds [18].

**Figure 4:**
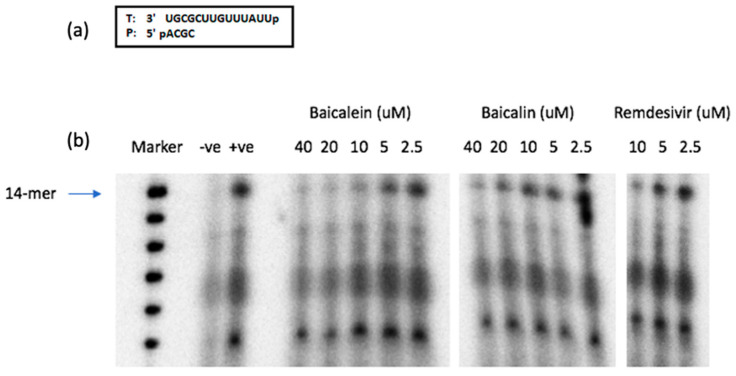
Inhibition of RNA-dependent RNA polymerase activity by baicalein, baicalin, and RDV-TP. (**a**) RNA 4-mer primer/14-mer template used in the RNA polymerase reaction. (**b**) Full-length 14-mer RNA product synthesis by SARS-CoV-2 polymerase complex (nsp12/nsp7/nsp8) in the presence/absence of indicated concentrations of each compound. (-ve) is the control without RdRp complex. (+ve) is the no-treatment control for the reaction. (Marker) is the molecular size marker.

Shown in Figure 4, baicalein demonstrated more potent inhibition of SARS-CoV-2 RdRp activity compared to baicalin, as observed by the lower amount of the 14-nucleotide RNA product generated, especially at the higher compound concentrations tested. The nucleotide analog remdesivir-triphosphate (RDV-TP) which was previously shown to be an effective inhibitor of SARS-CoV-2 RdRp [14,18], was used as a control in this assay. In fact, the levels of suppression of RNA polymerase activity were more prominent with baicalein than with the RDV-TP. Unlike RDV-TP, which causes delayed chain-termination during RNA synthesis via incorporation of the nucleotide analog by the RdRp [18], we hypothesize that these flavonoids exert their inhibitory effects on the virus polymerase activity via different mechanism(s) of action such as binding to the SARS-CoV-2 RdRp.

**Figure 5:**
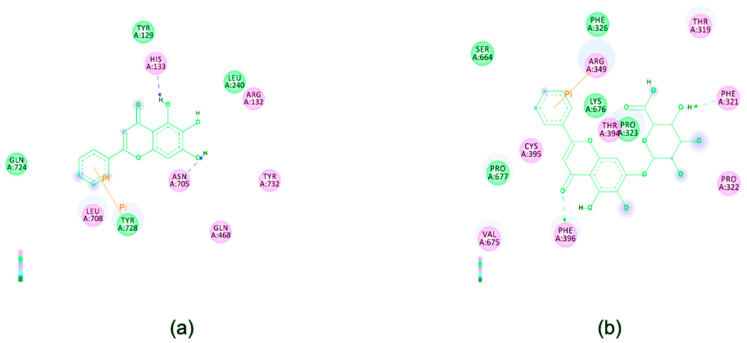
Illustrations of interactions between (**a**) baicalein and (**b**) baicalin and SARS-CoV-2 RdRp. 2D diagrams of ligand–protein interactions showing the amino acid residues involved were generated by Discovery Studio 2.5.

As these two compounds are not nucleoside analogs, the most probable mechanism of inhibition might be through binding to the SARS-CoV-2 RdRp in a place other than the active site. Therefore, we performed an in silico study to identify potential binding between the tested compounds and SARS-CoV-2 RdRp. Our data showed that both compounds could strongly bind to SARS-CoV-2 RdRp with a binding energy of −8.7 and −7.8 kcal/mol, respectively, in different positions (Figure 5). Interestingly, our in silico data also showed that baicalein could hypothetically bind to His133 and Asn705 amino acids in SARS-CoV-2 RdRp which are found in the nucleotidyltransferase domain and the palm subdomain of the RdRp, respectively [24]. While the highly conserved palm subdomain forms part of the enzyme’s active site [25,26], the actual role of the NiRAN domain remains unclear. Nonetheless, the importance of the NiRAN domain towards successful virus replication is evident, as nucleotide substitution in this segment of the RdRp interrupted the replication capacities of equine arteritis virus and SARS-CoV [27]. In comparison with a previous study, both flavonoids appeared to exhibit stronger binding energies against SARS-CoV-2 RdRp than that of remdesivir (−6.5 kcal/mol) [28]. The similar study also reported remdesivir’s specific binding sites on the virus RdRp that differ from our present findings with baicalein and baicalin. This indicates that the anti-SARS-CoV-2 activities of these flavonoids may involve different mechanism(s) of action than that of remdesivir. Hence, baicalein or baicalin have the potential to be considered as treatment options along with remdesivir, which is currently the main therapeutic agent for SARS-CoV-2-infected patients in clinical settings.

As our SARS-CoV-2 RdRp assay data demonstrated that baicalein is a more potent inhibitor of SARS-CoV-2 RdRp, we performed a thermal shift assay (TSA) to assess the binding of baicalein to different SARS-CoV-2 nonstructural proteins. Our data showed that baicalein, even at 50 µM, did not appear to have a significant effect on the stability of either SARS-CoV-2 nsp5 or nsp14, suggesting that this compound does not bind these two proteins (Table 3). A previous report showed that baicalin can inhibit the enzymatic activity of SARS-CoV-2 3CL protease (nsp5) with an IC_50_ = 34.7 µM, which is much higher than the EC_50_ that we report here from baicalin against SARS-CoV-2 [29]. However, they did not provide any data regarding baicalein in their report. Liu and colleagues through a molecular docking study showed that baicalein can also hypothetically bind to the N- and C-terminal active pockets of the SARS-CoV-2 nsp14 [30]. However, they did not provide any laboratory-based data as a proof of concept for their hypothesis. Su and colleagues also showed the antiprotease activity from baicalein and baicalin against SARS-CoV-2 3CL with IC_50_ values of 0.94 and 6.41 µM, respectively, based on their enzyme-inhibition experiments [31]. However, they reported the EC_50_ values of 2.94 and 27.87 µM for baicalein and baicalin, respectively, in Vero E6 cells. Based on our data, baicalein appeared to have a more stabilizing effect on nsp12 (Table 3), suggesting that this compound binds to nsp12, which is supported by the RdRp and in silico data. Therefore, all abovementioned findings could suggest that baicalein and baicalin may have dual effects on SARS-CoV-2 RdRp and 3CL protease, which could be interesting for further investigation and is worth consideration for future studies.

Antiviral activity of baicalein and baicalin against a wide range of different viruses has been reported, including several enveloped RNA viruses such as influenza, dengue, chikungunya, zika viruses [4,5,6,7,8] and also some non-enveloped RNA viruses such as enteroviruses [32]. However, the exact mechanism(s) of action for those compounds against different viruses is yet to be elucidated, even though Lalani and colleagues showed the extracellular activity of baicalein against enterovirus 71 (EV-71) [32]. Here, we reported the antiviral activity of both compounds against SARS-CoV-2 through binding to SARS-CoV-2 RdRp. However, this finding also needs further investigations in addition to a comprehensive mechanism study for the compounds against other viruses as well.

## 4. Conclusions

In this study, we identified two natural products, baicalein and baicalin, as novel antiviral therapeutic candidates that for the first time demonstrate SARS-CoV-2 RdRp inhibition, thus warranting further evaluation and characterization towards drug development for treatment of COVID-19. We have shown that both compounds could inhibit the SARS-CoV-2 RdRp through specific binding, while baicalein showed stronger binding affinity compared to baicalin. These compounds are natural bioactive polyphenolic flavonoid compounds and are widely used in traditional Chinese medicine as a remedy for the treatment of various medical conditions [9,10]. Based on the SARS-CoV-2 antiviral potency and the safety profile of these compounds, we propose further studies on these compounds and structurally related derivatives, alone and also in combination with effective nucleoside analogs such as remdesivir towards generating compelling data to advance an anti-COVID-19 drug candidate for further preclinical studies.

## Figures and Tables

**Table 1:** Anti-SARS-CoV-2 activity and cytotoxicity of selected compounds. Anti-SARS-CoV-2 activity of each compound evaluated in Vero and human Calu-3 cells; the cytotoxicity was also determined in separate experiments.

Compound	Antiviral Activity against SARS-CoV-2				Cytotoxicity	
	Vero		Calu-3		Vero	Calu3
	EC_50_ (µM)	EC_90_ (µM)	EC_50_ (µM)	EC_90_ (µM)	CC_50_ (µM)	CC_50_ (µM)
Baicalein	4.5 ± 0.2	7.6 ± 0.3	1.2 ± 0.03	6.2 ± 0.04	8 6 ± 0.1	91 ± 0.05
Baicalin	9.0 ± 0.08	15.8 ± 0.2	8.0 ± 0.11	15.1 ± 0.2	>100	>100
Remdesivir	1.0 ± 0.1	3.3 ± 0.3	0.14 ± 0.02	0.5 ± 0.03	> 100	> 100

**Table 2:** SARS-CoV-2 pseudovirus entry assay. Different concentrations of each compound have been tested against entry stage of the SARS-CoV-2 pseudovirus. Pseudovirus entry inhibition (%) has been defined compared to the readout of untreated sample.

Compound	SARS-CoV-2 Pseudovirus Entry Inhibition (%)	Compound	SARS-CoV-2 Pseudovirus Entry Inhibition (%)
Baicalin, µM		Baicalein, µM	
30	−3.3	30	14.6
10	−6.6	10	3.9
3.3	−11.9	3.3	−1.9
1.1	−12.1	1.1	−2

**Table 3:** Thermal shift assay results. Binding of baicalein to SARS-CoV-2 RdRp (nsp12), 3CL protease (nsp5) and nsp14 as another important nonstructural protein of SARS-CoV-2 was tested. Baicalein caused a ΔTm of 3.9 °C of nsp12, demonstrating strong binding to the main component of the SARS-CoV-2 RdRp.

Experiment Components	*T_m_* (°C)	Δ*T_m_* (°C)
nsp5 + DMSO	43.7 ± 0.1	-
nsp5 + Baicalein	44.5 ± 0.0	0.9
nsp12 + DMSO	44.4 ± 0.4	-
nsp12 + Baicalein	48.3 ± 2.6	3.9
nsp14 + DMSO	41.1 ± 0.1	-
nsp14 + Baicalein	42.6 ± 0.1	1.5

## Data Availability

Data sharing not applicable.
